# Supplementary material for: Seminoma and Embryonal Carcinoma Footprints Identified by Analysis of Integrated Genome-Wide Epigenetic and Expression Profiles of Germ Cell Cancer Cell Lines
Source: PLoS One. 2014 Jun 2;9(6):e98330. doi: 10.1371/journal.pone.0098330 (PMC4041891; doi:10.1371/journal.pone.0098330)
Supplement: File S1 — ZIP file containing DMRforPairs output for significant regions. Please start from the html files. (ZIP) [file pone.0098330.s008.zip › figures/10396.pdf]

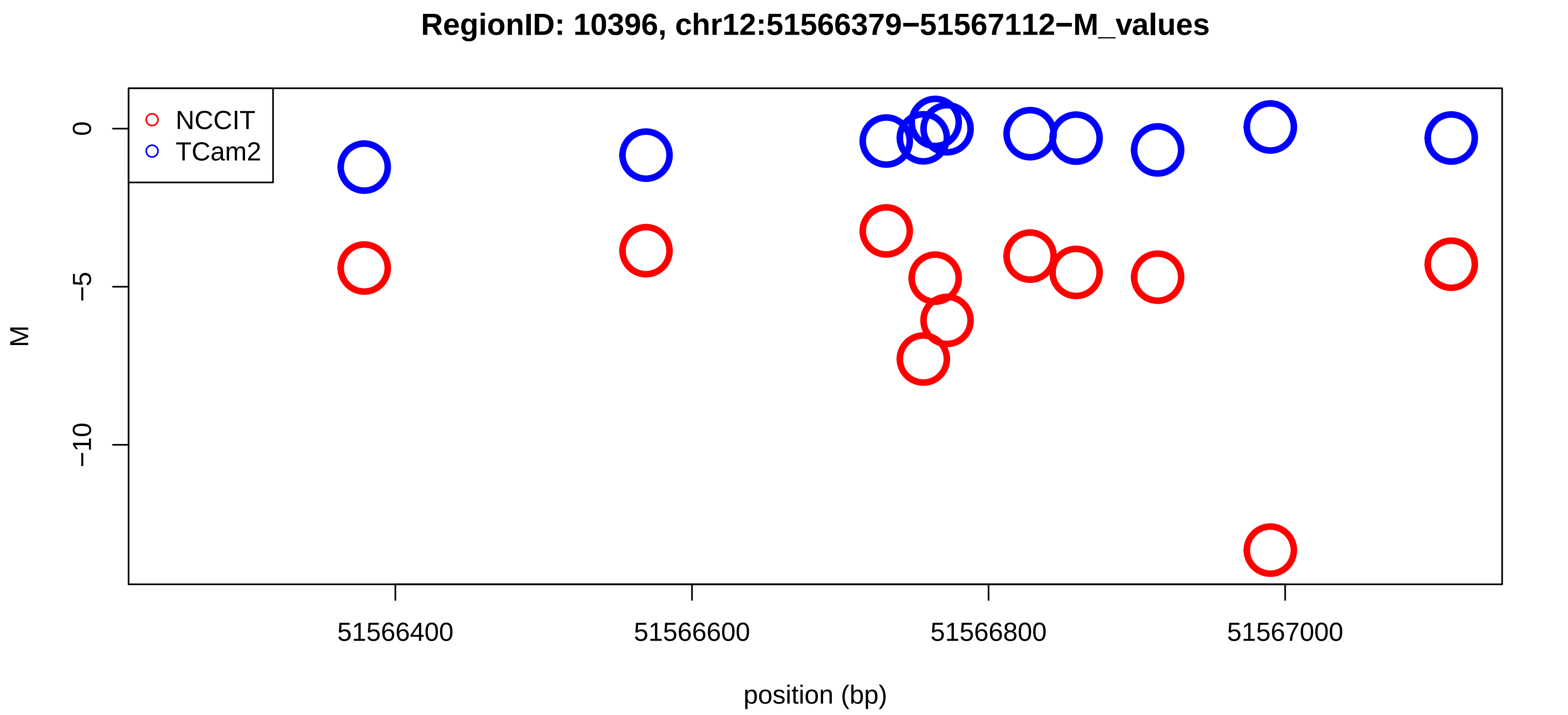
RegionID: 10396, chr12:51566379−51567112−M_values
NCCIT
TCam2
0
−5
−10
M
51566400
51566600
51566800
51567000
position (bp)

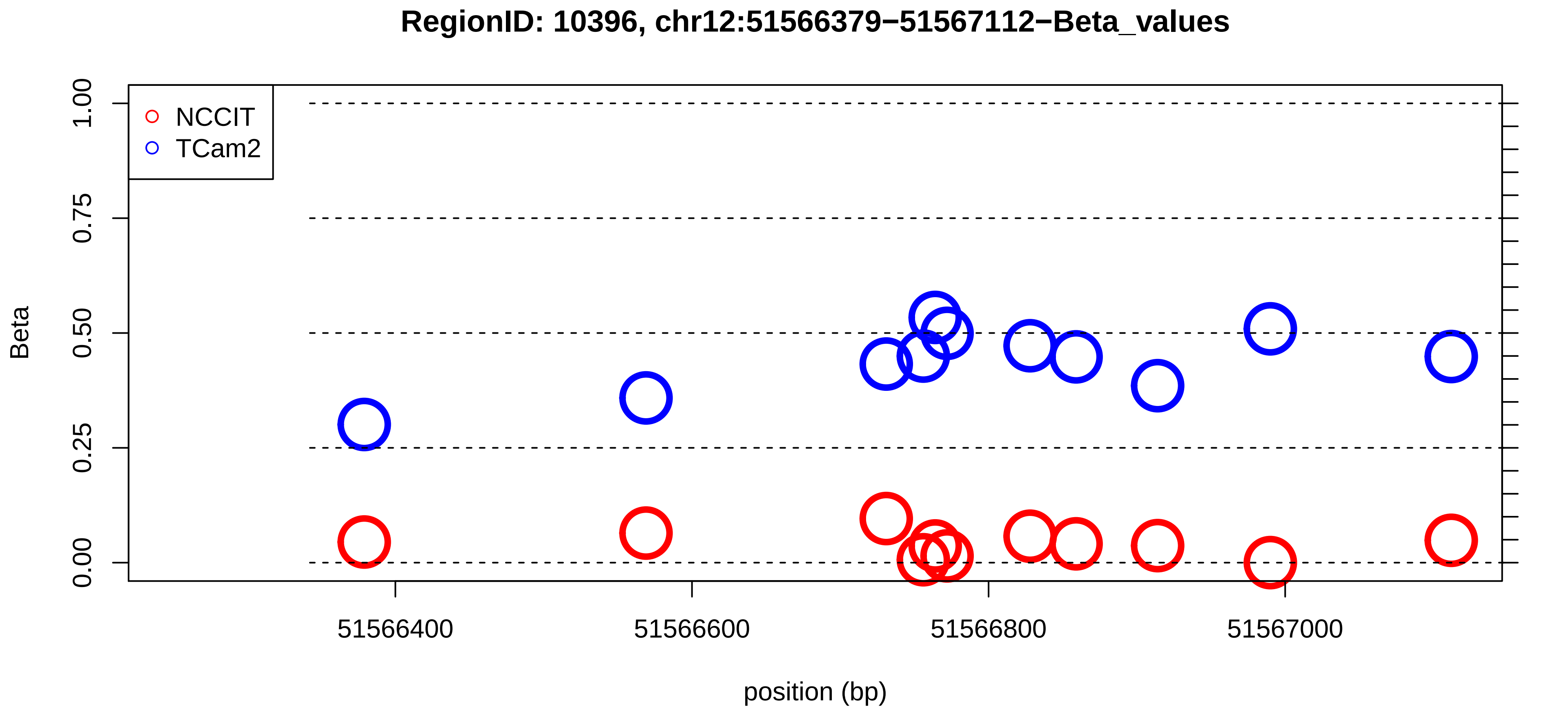
RegionID: 10396, chr12:51566379−51567112−Beta_values
NCCIT
TCam2
1.00
0.75
0.50
0.25
0.00
Beta
51566400
51566600
51566800
51567000
position (bp)

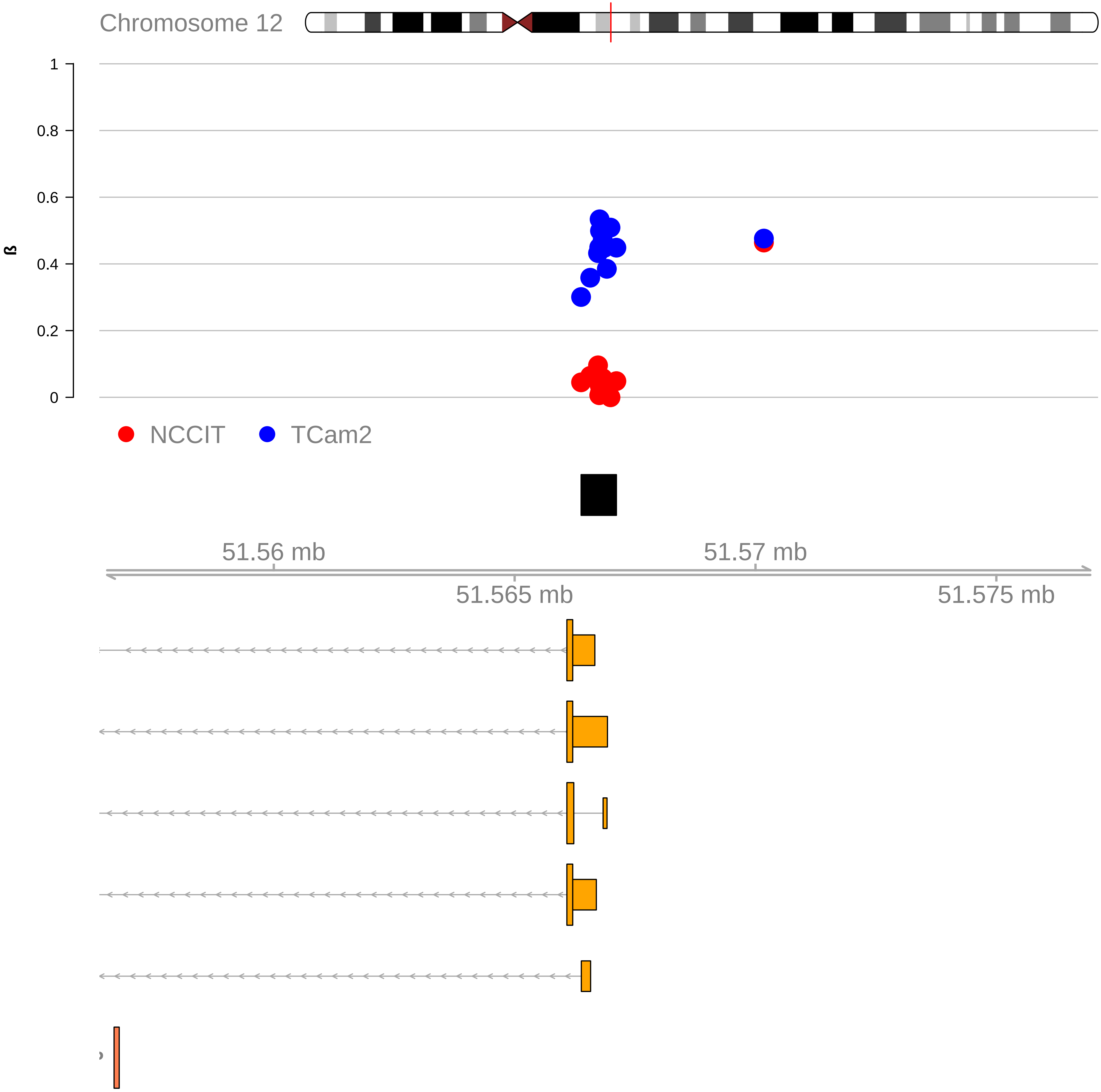

Chromosome 12
1
0.8
0.6
0.4
0.2
0
ß
NCCIT
TCam2
51.56 mb
51.57 mb
51.565 mb
51.575 mb
